# Knowledge, risk perceptions and attitudes of nurses towards HIV in a tertiary care hospital in Mangalore, India

**DOI:** 10.1186/1742-4690-9-S1-P68

**Published:** 2012-05-25

**Authors:** Anand Venugopal, A Basavaprabhu, B Unnikrishnan

**Affiliations:** 1 Radiology at Kasturba Medical College Hospital, Mangalore, India

## Introduction

Infectious diseases like HIV are on rise in developing countries like India which puts heavy burden on health care needs. Nurses have a key role and spend considerable time taking care of HIV positive patients admitted in hospitals. Hence a study was conducted in our hospital to have an insight into their knowledge about HIV, their apprehensions while taking care of such patients and their attitudes and willingness to take care of them.

## Methods

It’s a cross sectional study done among 200 nurses of KMC Hospital, Mangalore. They were given validated questionnaire comprising of 67 items which included knowledge of spread of HIV, universal precautions, risk perceptions and their attitudes towards HIV positive patients. Their responses were analysed using SPSS software.

## Results

Of the 200 Nurses selected, 152 completed the questionnaire. Regarding knowledge of HIV transmission, the correct response for widely advertised modes of transmission were higher-sexual contact 97.4%, vertical transmission 88.8%. However, 11.2% did not know about mother to child transmission and 28.9% about transmission by breast feeding. 90.1% felt HIV could be transmitted by sharing of plates and 83.6% felt by mosquito bites.93.4% knew about universal precautions and 78.3% routinely practiced it. 80.3% were aware of post exposure prophylaxis.79.2% described caring HIV Positives as rewarding,86.5% were willing to assist operations on hiv patients and 84.9% were willing conduct deliveries. 13.4% felt they have right to refuse caring HIV patients and 97% felt surgical patients need to be routinely tested for HIV.

## Conclusions

This study demonstrates that there are deficiencies in knowledge about HIV and false beliefs exist among nurses about spread of HIV. There is need to improve awareness about HIV and HIV patient care through training programmes to clear misconceptions amongst Nurses so that HIV positive patients are not discriminated against and are treated without discrimination.

